# Assessing We-Disease Appraisals of Health Problems: Development and Validation of the We-Disease Questionnaire

**DOI:** 10.3390/ejihpe14040061

**Published:** 2024-04-03

**Authors:** Alexandra J. Vogt, Lasse Bartels, Isabella C. Bertschi, Fiona Mahler, Michael Grotzer, Daniel Konrad, Kurt Leibundgut, Jochen Rössler, Guy Bodenmann, Markus A. Landolt

**Affiliations:** 1 Division of Child and Adolescent Health Psychology, Department of Psychology, University of Zurich, 8050 Zurich, Switzerland; 2 Department of Psychosomatics and Psychiatry, University Children’s Hospital Zurich, University of Zurich, 8032 Zurich, Switzerland; 3 Children’s Research Centre, University Children’s Hospital Zurich, University of Zurich, 8032 Zurich, Switzerland; michael.grotzer@kispi.uzh.ch (M.G.);; 4 Division of Clinical Psychology for Children/Adolescents and Couples/Families, Department of Psychology, University of Zurich, 8050 Zurich, Switzerlandguy.bodenmann@psychologie.uzh.ch (G.B.); 5 Department of Paediatric Endocrinology and Diabetology, University Children’s Hospital Zurich, University of Zurich, 8032 Zurich, Switzerland; 6 Division of Pediatric Hematology/Oncology, Department of Pediatrics, University Hospital Berne, 3010 Berne, Switzerland; k.leibundgut@gmail.com (K.L.); jochen.roessler@insel.ch (J.R.)

**Keywords:** we-disease appraisals, health impairment, chronic illness, couple relationship, dyadic coping, we-disease

## Abstract

In couples dealing with health problems, we-disease appraisals can influence dyadic coping strategies to alleviate distress. This study describes the development and validation of a self-report scale to assess we-disease appraisals of health problems. The newly developed We-Disease Questionnaire (WDQ) was administered in three samples: parents of children with type 1 diabetes (*n* = 240) or cancer (*n* = 125) and individuals with visual impairment and their partners (*n* = 216). Reliability was measured by coefficient omega. To assess construct validity, correlations with other measures of individual and dyadic adjustment were examined. Descriptive statistics across all samples were compared. A 4-item version of the WDQ demonstrated good reliability and validity and showed meaningful associations with established scales. We-disease appraisals were highest among parents of children with cancer and lowest among couples with visual impairment. The WDQ is a reliable and valid measure that can be used across different health problems.

## 1. Introduction

Individuals facing severe health impairments such as chronic illnesses or disabilities often experience significant psychological distress [1]. However, a severe illness can also evoke substantial distress in close-relationship partners. For instance, in a previous study, two in five cancer caregivers showed clinical levels of depression, and almost half of them reported clinically relevant symptoms of anxiety [2].

Similarly, a child’s impaired health can be detrimental to the parents’ psychological well-being. Parents of chronically ill children have been shown to be at a significantly higher risk of developing depression and anxiety compared to parents of healthy children [3], and they often exhibit symptoms of post-traumatic stress [4]. Moreover, marital adjustment can be poor in parent couples with a cumulation of stressors [5].

While a significant number of individuals and couples are negatively affected when their child or one of the partners becomes ill, many individuals and couples also cope well and do not report prolonged levels of elevated distress [6].

One major dyadic resource is dyadic coping. Dyadic coping encompasses couples’ interactions in dealing with stressors, i.e., supportive actions from one partner to the other and conjoint coping efforts involving both partners [7]. Conjoint forms of dyadic coping are most strongly and most consistently linked to relational functioning in community samples [8]. Conjoint dyadic coping encompasses “responses to stress experienced by both partners and/or to problems that partners see as sharing (“our” problem) even if they originated in one partner” [9] (p. 13). Conjoint forms of dyadic coping have proven beneficial for individual and dyadic adjustment in couples coping with one partner’s cancer [10], type 2 diabetes [11], or their child’s illness [12].

Several theoretical frameworks suggest that appraisals of the health problem as a shared stressor for the couple could influence how strongly couples engage in dyadic coping and what forms of dyadic coping they engage in e.g., [13,14]. It is also part of the concept of “we-disease” (WD) [14], where both partners appraise the illness as a shared problem and joint challenge, with which they have to deal together in the sense of common/joint dyadic coping.

So far, three explicit approaches to assess dyadic appraisals have been reported: single items that assess ownership or coping responsibility with regards to the health problem e.g., [15], trained coders’ assessments of couples’ conversations e.g., [16], and assessing dyadic appraisals using a proxy variable e.g., [17]. Furthermore, shared appraisals were assessed in diary studies e.g., [18]. While these approaches have generated important insight into the relevance of dyadic appraisals, they have several shortcomings, e.g., a lack of nuance in respondents’ answers, with single-item choices or high costs for behavioural coding. Thus, the field would benefit from a validated scale that is short for use in clinical samples and allows for gradual responses.

To our knowledge, no validated scale is currently available for assessing WD appraisals in couples coping with health problems in their partners or in their children. In the present study, we describe the development and initial validation of such a scale, the We-Disease Questionnaire (WDQ), in three samples of couples coping with health problems.

The overall goal of the present study was to develop a brief scale—the WDQ—assessing WD appraisals and to evaluate its psychometric properties. The first aim was to investigate the factor structure of the scale. Considering the goal of developing a brief scale assessing WD appraisals, we first hypothesized that items of the scale load on one overarching factor of WD appraisals. Second, we aimed to investigate the reliability of the scale across different samples. We hypothesized that the scale shows good reliability across different samples. Lastly, we aimed to investigate the construct validity of the scale. We hypothesized that the newly developed WDQ shows significant correlations with established scales.

## 2. Methods

### 2.1. Sample Procedures

#### 2.1.1. Study 1

Data on parent couples of children with type 1 diabetes (T1D) were taken from a study conducted at the University Children’s Hospital Zurich that aimed to investigate risk factors of metabolic control in children and adolescents with T1D. It received ethical approval from the local Ethics Committee (BASEC-ID: 2018-00374). Families were eligible for participation if the child was between 7 and 18 years old, had been diagnosed with T1D at least one year prior to data collection, and did not present with any medical comorbidities affecting glycemic control. Eligible families from the hospital database were informed about the study during regular follow-up visits. Upon written consent, the study team made an interview appointment within two weeks. Families did not receive financial compensation for participation.

The sample of this study consisted of 127 mothers and 113 fathers (*N* = 240). Mean age of mothers was 45.8 years (*SD* = 5.2, range = 31–58), and mean age of fathers was 48.5 (*SD* = 5.8, range = 32–65); 54% of all participants reported having completed at least some tertiary education; 67% of mothers and 95% of fathers were employed at the time of the survey. Average workload for paid work was 60% for employed mothers and 97% for employed fathers. Brief descriptions of overall goals and determinations of sample sizes for each individual study are available in the Supplementary Materials.

#### 2.1.2. Study 2

Data on parent couples of children with cancer were taken from a study conducted at the University Children’s Hospital of Zurich and the University Hospital of Berne that aimed to investigate the longitudinal influence of parental factors on quality of life in children with recently diagnosed cancer. It received ethical approval from the local Ethics Committee in Zurich (ID: 2015-0086) and Berne (ID: 067/2015). Children were eligible for participation if they were between 0 and 17 years old and had been newly diagnosed with cancer. Both partners gave written informed consent. Families did not receive financial compensation for participation.

The sample consisted of 65 mothers and 60 fathers (*N* = 125) assessed at 3 to 6 weeks after their child had been diagnosed with cancer. Mean age of mothers was 41.3 years (*SD* = 6.6, range = 30–60), and mean age of fathers was 44.1 (*SD* = 6.7, range = 30–64); 54% of all participants reported having completed at least some tertiary education; 62% of mothers and 97% of fathers were employed at the time of the survey. Average workload for paid work was 55% for employed mothers and 97% for employed fathers.

#### 2.1.3. Study 3

Data on couples in which one partner had a visual impairment were taken from a study conducted at University of Zurich that aimed to investigate the impact of visual impairment on couple relationships. It received ethical approval from the Ethics Committee of the Faculty of Arts and Social Sciences at the University of Zurich (approval #19.4.6). Couples were eligible for participation if one partner had a visual impairment that had developed or significantly deteriorated during the current relationship. Both partners gave written informed consent. Participants did not receive financial compensation for their participation.

A total of *N* = 216 individuals from 114 mixed-gender couples participated. Of these, 110 were individuals with visual impairment (IVI) (females: 50%), and 106 were partners of individuals with visual impairment (IVI) (females: 52%); 30% of IVIs reported experiencing markedly impaired vision for up to nine years, 31% for 10 to 19 years, and 39% for 20 years or more.

Mean age was 58.7 years (*SD* = 16.3, range = 31–93) for IVIs and 60.1 years (*SD* = 15.5, range = 28–89) for partners; 49% of participants reported having completed at least some tertiary education. Among IVIs, 27% were employed at the time of data collection, 28% were retired, 35% received a full disability pension, and 9% were employed and received partial disability pension. Among partners, 51% were employed, 42% were retired, and 7% received a full disability pension.

### 2.2. Measures

#### 2.2.1. We-Disease Appraisal Items

The We-Disease Questionnaire (WDQ) was developed by one of the authors, GB, an expert in dyadic coping with extensive clinical training and experience in couple and family therapy. The items were generated along two dimensions (shared/non-shared; problem-oriented/emotion-oriented) according to the Systemic Transactional Model (STM) [7]. The objective from the outset was to develop a short scale for clinical application, minimizing respondent burden. An initial set of 20 items was created, evaluating the perception of the health problem as a collective stressor, the similar emotional and practical effects of the disease, and the implications for interpersonal relationships. These items represented three dimensions: (a) who is the carrier of the disease, (b) who is affected in which way by the disease, and (c) who has what resources to contribute to common dyadic coping. The focus of these items was on appraisal rather than concrete behaviors. Subsequently, an expert panel consisting of two senior researchers and one junior researcher methodically evaluated, rated, and refined these items. The goal was to create a very brief questionnaire, not exceeding eight items. Finally, seven items were retained for validation after several rounds of critical review by this expert group.

The items can be used across a wide variety of health problems, and they can be accommodated for different couple types, e.g., parent couples of ill children or couples with one ill partner. An initial set of seven items was generated and consists of statements on WD appraisals of a health problem (see Table 1). Items are scored on a 6-point Likert scale ranging from 0 (*strongly disagree*) to 5 (*strongly agree*). The WDQ was administered in all three studies.

#### 2.2.2. Validation Measures

The WDQ was validated against selected well-established measures in the field of individual and dyadic adjustment to impaired health that had been administered in at least one of the studies.

**Dyadic Coping.** The Dyadic Coping Inventory (DCI) [19] is a 37-item self-report questionnaire measuring dyadic coping behaviors. We used the common dyadic coping (CDC) subscale and the total score of dyadic coping by one’s partner (DCPAR), calculated by the mean of all partner-related items of the DCI (item 5–19). The CDC subscale measures conjoint efforts of the partners to cope when they are both feeling stressed with 5 items. The scale ranges from 1 = “very rarely” to 5 = “very often”. DCPAR combines all facets of dyadic coping the respondent receives from their partner. The DCI subscales and total scores have shown good internal consistency in a validation study [20] and in the samples from study 2 (CDC: McDonald’s Omega ω = 0.79, DCPAR: ω = 0.90) and study 3 (CDC: ω = 0.76, DCPAR: ω = 0.87).

**Relationship Quality.** Relationship quality was assessed using the short version of the Partnership Questionnaire (PFB-K) [21]. The PFB-K has three subscales measuring tenderness, conflict behavior, and togetherness/communication with three items each. All subscale items are rated from 0 = “never/very rarely” to 3 = “very often”. Internal consistency for the total score was high in the original validation study [21] and in the sample from study 3 (ω = 0.83).

**Intimacy.** Intimacy was assessed using four items developed to assess intimacy in a daily diary study [22]. The scale consisted of four items, rated on a 5-point scale from 0 = “not at all true” to 4 = “very true”. Internal consistency was good in the original study and in the sample from study 3 (ω = 0.85).

**Depressive Symptoms.** Depressive symptoms were assessed using the 7-item depression subscale of the 21-item version of the Depression Anxiety Stress Scale (DASS-21) [23]. The 4-point scale was simplified for the present study and ranged from 0 = “not at all” to 3 = “very much”. The German version of the DASS-21 depression subscale has shown excellent internal consistency in patients with health problems [24]. Internal consistency was also good in the sample from study 3 (ω = 0.86).

### 2.3. Statistical Analyses

As mentioned above, a one-factor structure was presumed while developing a short and clinical useful instrument to assess WD appraisals. Thus, confirmatory factor analysis (CFA) was preferred over exploratory factor analysis to test the WDQ’s factor structure [25]. The aim was to develop a clinical instrument that allows to assess the construct of WD appraisals in a quick and practicable manner for mental health professionals across various settings. Therefore, one general factor was expected due to the small number of items, and a CFA was utilized to investigate the factor structure of the scale. 

To assess the WDQ’s factor structure in parents of ill children, the samples from studies 1 and 2 were combined. Subsequently, we conducted CFAs on couples coping with one partner’s health impairment using data from study 3. CFAs were conducted in Mplus 8.5 [26]. Robust diagonally weighted least squares estimator (WLSMV) was used to account for categorical data and non-normality and was compared to robust maximum likelihood estimator (MLR) to check for robustness of CFA results [27]. In the dyadic CFAs, we included two latent factors representing mothers’ and fathers’ WD appraisals or IVIs and their partners’ WD appraisals. As recommended for CFA with dyadic data, the latent factors as well as the residual covariances across each pair of items were correlated [28]. To allow for variation based on role (mothers vs. fathers, IVIs vs. partners), factor loadings for corresponding item responses across partners were not constrained to be equal. Model fit in CFAs was evaluated using the following fit indices: chi-squared test of exact fit (χ^2^), comparative fit index (CFI), the standardized root mean square residual (SRMR), and the root mean square residual of approximation (RMSEA). We used the recommended ratio of χ^2^/*df* < 3 as an indicator for acceptable model fit alongside the following cut-offs for fit indices: CFI ≥ 0.95, SRMR ≤ 0.10, RMSEA ≤ 0.10 and CI for RMSEA close to its point estimate [29,30,31].

To assess reliability of the WDQ, robust estimates of McDonald’s coefficient omega using bias-corrected bootstrap confidence intervals [32] were calculated with R package “coefficientalpha”, version 0.7 [33]. Kruskal–Wallis test and post hoc Dunn’s test with Benjamini–Hochberg adjustment assessed differences in WD appraisals between all three samples. Paired samples *t*-tests were conducted to check for differences in WD appraisals between mothers and fathers in both parent samples and between IVIs and partners. To further assess validity, Pearson correlations between WDQ total scores and scale scores of the validation measures were calculated separately for mothers and fathers and for IVI and partners. Calculations were conducted using R version 4.0.3 [34] in RStudio Version 1.4.1103 [35].

## 3. Results

### 3.1. Factor Structure of the WDQ

**Figure 1:**
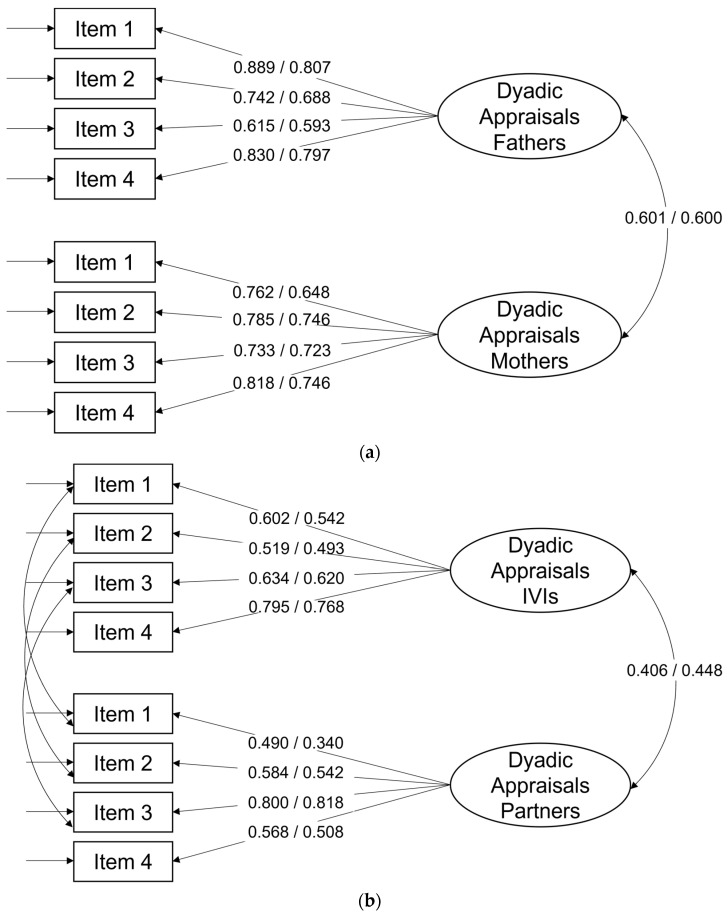
(**a**) Standardized factor loadings of WDQ items in parents of ill children; (**b**) standardized factor loadings of WDQ items in couples coping with one partner’s visual impairment. Note: values left of / refer to factor loadings estimated with WLSMV estimator; values right of / refer to factor loadings estimated with MLR estimator.

The initial 7-item dyadic CFA did not show a good fit of the data among parents of ill children (see Table 2 with Models 1.1 and 1.2). The misfit could be attributed to lower factor loadings on items 5, 6, and 7. Items 5 and 7 referenced a preference for individual over dyadic coping in respondents. It was thus decided to drop the two items for the sake of uniformity of the scale. The resulting 5-item version (Models 2.1 and 2.2) fitted the data better. However, item 6 still did not load as highly on the latent factors as the other four items (factor loadings ≤ 0.40). Item 6 assessed the impact of the child’s illness on the parents’ couple relationship. It was thus conceptually somewhat different from items 1 to 4 and may not assess WD appraisals in a narrow sense. Thus, a shorter 4-item version of the WDQ was tested. Using WLSMV estimator (Model 3.1), the CFA results indicated good model fit: χ^2^(15) = 43.90, *p* < 0.001, χ^2^/*df* = 2.93, CFI = 0.98, SRMR = 0.04, RMSEA = 0.10 [0.06, 0.13]. Model 3.2, using MLR estimator, also indicated good model fit: χ^2^(15) = 32.95, *p* = 0.005, χ^2^/*df* = 2.20, CFI = 0.96, SRMR = 0.06, RMSEA = 0.08 [0.04, 0.11]. The 4-item version produced uniformly high factor loadings (>0.70) on all four items except for item 3, which had slightly lower factor loadings for mothers. The standardized factor loadings for models 3.1 and 3.2 are shown in Figure 1a.

In couples coping with visual impairment, the initial 7-item dyadic CFA did not show a good fit of the data either (see Table 2 with Models 4.1 and 4.2). The model fit for the 5-item version without items 5 and 7 (Models 5.1 and 5.2) was much better. However, item 6 did not load as highly on the latent factors as the other four items (factor loadings ≤ 0.43). The CFA results of the shorter 4-item version with items 1–4 indicated good model fit: χ^2^(15) = 23.49, *p* = 0.074, χ^2^/*df* = 1.57, CFI = 0.97, SRMR = 0.04, RMSEA = 0.07 [0.00, 0.12]. Model 6.2, using MLR estimator, also indicated good model fit: χ^2^(15) = 23.94, *p* = 0.066, χ^2^/*df* = 1.60, CFI = 0.93, SRMR = 0.06, RMSEA = 0.07 [0.00, 0.12]. The 4-item version produced factor loadings ranging between 0.51 and 0.82. However, item 2 had lower loadings for IVIs, and item 1 had lower loadings for partners. The standardized factor loadings for models 6.1 and 6.2 are shown in Figure 1b.

### 3.2. Descriptive Statistics of the WDQ

Mean scores across the four retained items from WDQ were calculated to obtain a WD appraisal score for respondents and compare the samples (*H*(2) = 108, *p* < 0.001). Post hoc tests showed that WD appraisal scores were higher in parents of children with cancer than in parents of children with T1D, *U*(*N*_study 2_ = 125, *N*_study 1_ = 240) = −8.44, *p* < 0.001, and than in couples coping with one partner’s visual impairment, *U*(*N*_study 2_ = 125, *N*_study 3_ = 226) = −10.10, *p* < 0.001. WD appraisal scores were also higher in parents of children with T1D compared to couples coping with one partner’s visual impairment, *U*(*N*_study 1_ = 240, *N*_study 3_ = 226) = −2.17, *p* = 0.03. Furthermore, in couples coping with one partner’s visual impairment, men reported significantly higher WD appraisal scores than women, but only among IVIs *U*(*N*_women_ = 55, *N*_men_ = 55) = −2.63, *p* = 0.010 and not among partners *U*(*N*_women_ = 56, *N*_men_ = 50) = −0.02, *p* = 0.987. Detailed information regarding group differences can be found in Table S1 in the Supplementary Materials.

### 3.3. Psychometric Properties of the WDQ

#### 3.3.1. Reliability

Reliability of the 4-item WDQ was good, with ω = 0.78 [0.61, 0.85] for mothers of a child with cancer, ω = 0.79 [0.71, 0.84] for mothers of a child with T1D, and ω = 0.81 [0.72, 0.86] for fathers of a child with T1D. However, reliability was lower in fathers of a child with cancer, ω = 0.53 [0.27, 0.72]. In study 3, reliability of the WDQ was also good, as indicated by ω = 0.70 [0.57, 0.79] for IVIs and ω = 0.66 [0.49, 0.74] for partners. Reliability was comparable in women, ω = 0.66 [0.50, 0.77], and in men, ω = 0.69 [0.57, 0.77].

#### 3.3.2. Construct Validity

Investigating the convergent validity, the results showed moderate positive associations between WDQ mean scores and CDC and DCPAR in parent couples of children with cancer (CDC: *r* = 0.29; DCPAR: *r* = 0.34) and couples coping with one partner’s visual impairment (CDC: *r* = 0.20; DCPAR: *r* = 0.21) (Table 3). WD appraisal scores were further positively associated with relationship quality (*r* = 0.21) and intimacy (*r* = 0.28). Evaluating the discriminant validity, the results showed no significant associations with depressive symptoms.

## 4. Discussion

The aim of the present study was to develop and psychometrically test the WDQ, a short scale to assess WD appraisals of a health problem as a shared stressor in couples. The WDQ is, to our knowledge, the first published self-report scale to measure health-related WD appraisals. The items have been developed on a theoretical basis [7] and consider a trade-off between requirements in research versus clinical practice. The reliability and validity of the WDQ have been tested in couples coping with their child’s illness and couples coping with one partner’s health impairment, as well as across three health problems with differing characteristics: cancer, T1D, and visual impairment (IVI).

The 4-item WDQ showed moderate-to-good internal consistency across the different samples. Construct validity of the WDQ was established by moderate associations with dyadic coping by one’s partner and with common/joint DC, as well as by moderate associations with relationship quality and intimacy. These findings are in line with theoretical work suggesting that WD appraisals relate to dyadic adjustment through dyadic coping [13]. Furthermore, WD appraisal scores were not associated with individual well-being (depression) as a distal outcome, supporting the discriminant validity of the WDQ.

Descriptive statistics indicated noteworthy group differences in WD appraisals based on contextual factors. WD appraisal scores were higher in parents of children with cancer than in parents of children with T1D. This could be due to differences in the respective illnesses’ salience as a stressor. Cancer represents an imminent threat to the child’s life, creates similar emotional distress for both parents, and requires immediate adaptations that affect the entire family. T1D, in contrast, poses a less imminent threat to the child’s life and may affect the parent who mainly cares for the child and supports the child in disease management more than the other parent. Differences in salience of the stressor could also explain why WD appraisals were lowest among couples coping with vision loss, a chronical impairment that brings about pronounced functional limitations without lethal risk. Furthermore, the fact that the couples included in this study had been suffering from vision impairment for many years might have eroded WD appraisals as an acute strategy for dealing with the illness or handicap. Those couples might already have largely adapted to living with restrictions due to the vision impairment. On the other hand, parents of a child with cancer were interviewed shortly after diagnosis, which made WD most salient. Again, parents of children with T1D diagnosis in study 2 were already somehow adapted to the illness, as it had occurred at least one year prior to data collection. Thus, WD might be most pronounced when illness is more recent and is demanding the mobilization of new strategies and common/joint DC being confronted to a novel and severe stressor.

The finding that no particular differences were found between parents dealing with child illness or between patients and partners in the IVI may support the notion of shared processes as assumed in STM. This suggests that the salience of the stressor and the time since the occurrence of the illness, as outlined above, may be most decisive for WD appraisals and less so for role differences (patient versus caregiver). Role differences in WD appraisals have, however, been reported in diabetes [36] and warrant attention in future research.

Gender is another contextual factor that may relate to WD appraisals. In IVI couples, men reported significantly higher WD appraisal scores than women, but only among IVIs and not among partners. One possible explanation could be that women have more mutually responsive relationships outside of romantic relationships than men [37]. That is, when confronted with a serious stressor such as a visual impairment, women might share their experiences with more close others and, consequently, appraise it to be less of a shared stressor within the couple relationship. However, while gender interacted with role to affect WD appraisals in study 3, no gender differences were found in studies 1 and 2 investigating parent couples. The latter finding suggests that parents are similarly engaged in their child’s illness and share appraisals regardless of their gender. In sum, further research is needed to disentangle the differential relevance of contextual factors (type of illness, stage of illness, duration since diagnosis, type of dyad (parent–child, couples etc.), and social embedment) for WD appraisals.

The WDQ seems to be generic enough to be used across different settings, health problems, and relationships in relation to the ill or impaired individual. Nonetheless, there are several limitations to this study. Firstly, not all originally formulated items were supported by the results of the factor analyses. Thus, future studies should assess whether there might be two distinct yet related factors: shared appraisals and non-shared appraisals. Secondly, two samples were merged for validation of the WDQ in parent couples. While this increased statistical power, it may have masked meaningful differences between the factor structures in each sample. However, the WDQ’s factor structure could be replicated in the sample from study 3, suggesting stability of the factor structure across populations. Thirdly, apart from the Dyadic Coping Inventory, the scales for evaluating construct validity were not administered across all subsamples. Thus, it was not possible to investigate construct validity across all samples. Therefore, interpretations of construct validity should be approached with caution, and future research should aim to establish construct validity across diverse populations. Lastly, we did not examine test–retest reliability of the WDQ, which should be rectified in future studies.

Studies in the context of other health problems are needed to further support the WDQ’s reliability, validity, and usefulness. For instance, it would be interesting to investigate WD appraisals in couples coping with health problems that have a high direct relationship impact, e.g., due to their interference with sexual function or communicative abilities. Furthermore, it would be interesting to compare WD appraisal scores in parent couples whose ill children belong to different age groups. Additionally, it is worthwhile to examine pathways that may explain how WD appraisals develop. Potential antecedents of WD appraisals may be found at the situational level (e.g., current stress levels), at the individual level (e.g., need for autonomy), and at the dyadic level (e.g., commitment). Another avenue for future research is congruence in WD appraisals. For instance, it has been argued that common/joint dyadic coping efforts are most likely in the case of high congruence in appraisals between partners [38], an assumption that could be readily tested using WDQ scores.

Developing WD appraisals and a “we-awareness” [39] should be central objectives of couple- and family-based psychosocial interventions that aim to foster adjustment to impaired health. The WDQ is well suited as a screening tool and can be used to identify couples or parents with low WD appraisals that need professional support and to assess effects of such interventions.

WD appraisals may develop particularly well when partners mutually share their individual experiences and are responsive to each other’s self-disclosure, which in turn is linked to positive individual and dyadic outcomes via dyadic coping [13]. Consequently, the settings couples navigate should acknowledge their being an entity (as a we). In healthcare and related settings, it is thus paramount to de-emphasize opposing roles and associated role expectations, e.g., the “patient” receives treatment and support while the “partner” is expected to provide support. Instead, a more holistic view of couples dealing with severe illness is needed where both are seen as affected by the stressors but also where both have resources to deal with them together [40]. This can be accomplished by including partners and family members in consultation or actively stimulating discussion about the impact of the health problem on the couple and family system.

## 5. Conclusions

The current study provides preliminary evidence that the WDQ is a reliable and valid measure of WD appraisals that has the potential to be used across different health problems, settings, and different types of couples (parent vs. patient/partner couples). The strengths of the WDQ include its short length, which makes it appealing for use in research and clinical practice, and the fact that the scale has been validated in three different samples. Future research is needed to investigate the construct of WD appraisals, its measurement, and its relation to contextual factors. WD appraisals are a promising avenue of research in the field of dyadic coping with chronic health problems. Education about and fostering of WD appraisals have the potential to enhance psychosocial couple interventions.

## Figures and Tables

**Table 1:** Prompts and item wordings for WDQ items.

Item Number	Item Wording
	The illness of our child… My visual impairment…/My partner’s visual impairment…
1	…is a shared challenge for us as a couple.
2	…creates similar practical problems for both of us.
3	…emotionally affects us in a similar way.
4	…we see as “our problem”; we have to go through it together.
5 *	…is better coped with individually by my partner and me. (r)
6 *	…strengthens our relationship.
7 *	…is a challenge that each of us must deal with by ourselves. (r)

*Note.* * = Item was removed after confirmatory factor analyses; (r) = item inversely coded.

**Table 2:** Model fit for confirmatory factor analyses on different versions of the WDQ.

Model	Included Items	Estimator	χ^2^	*df*	*p*	χ^2^/*df*	CFI	SRMR	RMSEA [90% CI]
*Parent couples of ill children*									
Model 1.1	1–7	WLSMV	426.89	69	<0.001	6.19	0.84	0.09	0.16 [0.14, 0.17]
Model 1.2	1–7	MLR	175.87	69	<0.001	2.55	0.86	0.09	0.09 [0.07, 0.10]
Model 2.1	1–4, 6	WLSMV	64.37	29	<0.001	2.22	0.98	0.04	0.08 [0.05, 0.10]
Model 2.2	1–4, 6	MLR	51.16	29	0.007	1.76	0.95	0.06	0.06 [0.03, 0.09]
Model 3.1	1–4	WLSMV	43.90	15	<0.001	2.93	0.98	0.04	0.10 [0.06, 0.13]
Model 3.2	1–4	MLR	32.95	15	0.005	2.20	0.96	0.06	0.08 [0.04, 0.11]
*Couples coping with visual impairment*									
Model 4.1	1–7	WLSMV	181.09	69	<0.001	2.62	0.80	0.09	0.12 [0.10, 0.14]
Model 4.2	1–7	MLR	127.78	69	<0.001	1.85	0.74	0.09	0.09 [0.06, 0.11]
Model 5.1	1–4, 6	WLSMV	41.31	29	0.065	1.42	0.97	0.05	0.06 [0.00, 0.10]
Model 5.2	1–4, 6	MLR	33.67	29	0.252	1.16	0.97	0.06	0.04 [0.00, 0.08]
Model 6.1	1–4	WLSMV	23.49	15	0.074	1.57	0.97	0.04	0.07 [0.00, 0.12]
Model 6.2	1–4	MLR	23.94	15	0.066	1.60	0.93	0.06	0.07 [0.00, 0.12]

*Note.* Item wordings can be found in Table 1. CFI = comparative fit index; SRMR = standardized root mean square residual; RMSEA = root mean square error of approximation; CI = confidence interval.

**Table 3:** Correlations between WDQ scores and other variables.

Variable	Parent Couples of Children with Cancer				Couples Coping with One Partner’s Visual Impairment					
	ω	Mothers (*n* = 65)	Fathers (*n* = 60)	Total Sample (*N* = 125)	ω	IVIs (*n* = 110)	Partners (*n* = 106)	Women (*n* = 111)	Men (*n* = 105)	Total Sample (*N* = 216)
Total dyadic coping by partner	0.90	0.46 ** [0.25, 0.64]	0.16 [−0.10, 0.39]	0.34 ** [0.18, 0.49]	0.86	0.33 ** [0.15, 0.48]	0.08 [−0.11, 0.27]	0.18 [−0.01, 0.35]	0.23 * [0.04, 0.41]	0.21 ** [0.08, 0.33]
Common/joint dyadic coping	0.79	0.36 ** [0.13, 0.55]	0.13 [−0.13, 0.38]	0.29 ** [0.12, 0.44]	0.76	0.25 ** [0.07, 0.42]	0.15 [−0.05, 0.33]	0.10 [−0.08, 0.29]	0.30 ** [0.12, 0.47]	0.20 ** [0.07, 0.32]
Relationship quality	−	−	−	−	0.83	0.23 * [0.05, 0.40]	0.18 [−0.01, 0.36]	0.13 [−0.05, 0.31]	0.31 ** [0.13, 0.48]	0.21 ** [0.07, 0.33]
Intimacy	−	−	−	−	0.85	0.32 ** [0.14, 0.48]	0.26 ** [0.07, 0.43]	0.22 * [0.03, 0.39]	0.34 ** [0.16, 0.50]	0.28 ** [0.15, 0.40]
Depressive symptoms	−	−	−	−	0.86	0.11 [−0.08, 0.29]	−0.04 [−0.23, 0.16]	0.10 [−0.09, 0.28]	−0.01 [−0.20, 0.18]	0.05 [−0.09, 0.18]

*Note*. ω = McDonald’s Omega (internal consistency); DC = dyadic coping; dashes indicate unavailable data; values in square brackets indicate the 95% confidence interval for each correlation; * indicates *p* < 0.05; ** indicates *p* < 0.01.

## Data Availability

The data presented in this study are openly available in FigShare at https://figshare.com/projects/Assessing_We-Disease_Appraisals_of_Health_Problems_Development_and_Validation_of_the_We-Disease_Questionnaire/189867.

## Supplementary Materials

The following supporting information can be downloaded at: https://www.mdpi.com/article/10.3390/ejihpe14040061/s1; Table S1: Descriptive statistics and group comparison of WDQ scores; STROBE cross-sectional checklist.
