# Supplementary material for: Assessing We-Disease Appraisals of Health Problems: Development and Validation of the We-Disease Questionnaire
Source: Eur J Investig Health Psychol Educ. 2024 Apr 3;14(4):941–53. doi: 10.3390/ejihpe14040061 (PMC11049654; doi:10.3390/ejihpe14040061)
Supplement: Supplementary file 1 [file ejihpe-14-00061-s001.zip › Table S1.pdf]

Table S1

Descriptive statistics and group comparisons of WDQ scores

Sample	Women	Men	*t*	*p*
Parents of a child with T1D (study 1)				
n	127	113		
M_{WDQ} (*SD*)	3.32 (1.04)	3.32 (0.99)	0.92	.359
Parents of a child with cancer (study 2)				
n	65	60		
M_{WDQ} (*SD*)	4.13 (0.79)	4.32 (0.58)	1.44	.157
Couples coping with visual impairment (study 3)				
IVIs				
n	55	55		
M_{WDQ} (*SD*)	2.85 (1.05)	3.35 (0.96)	-2.63	.010
Partners				
n	56	50		
M_{WDQ} (*SD*)	3.12 (0.91)	3.13 (0.93)	-0.02	.987

Note. Possible range of WDQ mean score: 0-5. T1D = type 1 diabetes; IVIs = individuals with visual impairment
